# Impact of radiotherapy in chemical composition and mechanical properties of human cervical dentin: an *in vitro* study

**DOI:** 10.1590/1678-7757-2024-0279

**Published:** 2025-03-14

**Authors:** Renata Borges RODRIGUES, Allyne Jorcelino Daloia de CARVALHO, Bruna Vanessa FELIPE E SILVA, Paulo Cézar SIMAMOTO, Veridiana Resende NOVAIS

**Affiliations:** 1 Universidade Federal de Uberlândia Faculdade de Odontologia Área de Dentística e Materiais Odontológicos Uberlândia Minas Gerais Brasil Universidade Federal de Uberlândia, Faculdade de Odontologia, Área de Dentística e Materiais Odontológicos, Uberlândia, Minas Gerais, Brasil.; 2 Universidade Federal de Uberlândia Faculdade de Odontologia Área de Oclusão, Prótese Fixa e Materiais Odontológicos Uberlândia Minas Gerais Brasil Universidade Federal de Uberlândia, Faculdade de Odontologia, Área de Oclusão, Prótese Fixa e Materiais Odontológicos, Uberlândia, Minas Gerais, Brasil.

**Keywords:** Biomechanical phenomena, Dentin, Hardness tests, Radiotherapy

## Abstract

Ionizing radiation directly affects hard dental tissues, compromising the dental structure, which results in damage to dentin collagen fibers and impacts the integrity of the dentin-enamel junction (DEJ).

**Objective:**

To evaluate the effects of radiotherapy on the chemical composition and mechanical properties of human cervical dentin.

**Methodology:**

Ten third molars were divided into control/non-irradiated and irradiated groups (n=5). The irradiated teeth were subjected to in vitro radiotherapy with the following protocol: 1.8 Gy daily, five days per week for eight weeks, totaling 72 Gy. The dentin in the cervical region was evaluated for each group. The chemical composition was assessed using Fourier transform infrared spectroscopy (FTIR) and Raman spectroscopy, focusing on the mineral/matrix ratio (M:M), carbonate/mineral ratio (C:M), and amide I/amide III ratio. Amide I/CH2 ratio was used to assess collagen quality, as amide I reflects protein conformation and hydrogen bonding, while CH2 indicates side-chain vibrations with low sensitivity to molecular orientation. Nanohardness and elastic modulus were evaluated by instrumented indentation. Scanning electron microscopy (SEM) was used to assess the enamel’s morphology. Statistical analysis of each parameter was performed using a t-test.

**Results:**

The FTIR analysis showed statistically significant differences in the C:M ratio (p=0.004) and amide I/amide III ratio (p=0.007). Raman spectroscopy revealed significant differences in the M:M ratio (p<0.001), as well as in the amide I/amide III (p<0.001) and amide I/CH2 ratios (p<0.001). Additionally, nanohardness (p=0.04) and the elastic modulus (p=0.003) showed statistically significant differences. SEM images revealed sound dentin shows normal tissue organization, whereas irradiated dentin showed no clear limit between peri and intertubular dentin.

**Conclusions:**

Radiotherapy induced significant changes in dentin composition and mechanical properties, characterized by increased organic content and phosphate levels, reduced carbonate, and decreased nanohardness and elastic modulus. These findings highlight the adverse effects on dentin's structural integrity.

## Introduction

Radiotherapy is an effective ionizing radiation-based treatment for tumors but causes adverse reactions affecting the patients’ quality of life.^1^ Common side effects in the head and neck region include mucositis, hyposalivation, xerostomia, trismus, osteoradionecrosis, and radiation caries. Mucositis is usually an acute condition, while hyposalivation and xerostomia can be long-lasting.^2^Similarly, the risks of caries and osteoradionecrosis may persist throughout the patient’s life.^1,2^The increased risk of radiation-related caries has a multifactorial etiology, including direct changes in dental tissues caused by irradiation.^3-5^

Radiation-related caries differ from conventional caries in location, appearance, and progression, typically occurring in the cervical area, between the crown and root, cuspid, and incisal regions.^2^ Early enamel shear fractures can rapidly lead to enamel loss and dentin exposure.^2^ As cervical destruction progresses, the dental crown loses support and can fracture completely, exposing the root. The destruction, caused by radiation, of acinar cells in the salivary glands reduces salivary flow, leading to hyposalivation, xerostomia, and alterations in the oral microbiota.^1^ This condition, often exacerbated by frequent consumption of high-carbohydrate foods, significantly contributes to dental caries development.^1^

The direct effects of ionizing radiation damage are hard dental tissues, resulting in a fragile dental structure.^6^Such changes include dentin collagen fibril damage, degeneration of odontoblast processes, gap formation at the dentin-enamel junction (DEJ), and alterations that can be detected in microhardness values.^5,7-,9^The mechanical properties and micromorphology, particularly the crystal properties and chemical composition of irradiated teeth, undergo degenerative changes.^3^

The correlation between radiotherapy and dental structure alterations, especially cervical region caries in head and neck cancer patients, is not fully understood and, given the high impact of radiation caries on the cervical region, a specific study is important. This study aimed to evaluate the effects of radiotherapy on the chemical composition and mechanical properties of human cervical dentin. The null hypotheses evaluated were that (1) radiotherapy does not alter the chemical composition and (2) does not affect the mechanical properties of cervical dentin.

## Methodology

This study used the Helsinki Declaration guidelines and was approved by the Research Ethics Committee of the Federal University of Uberlândia (Protocol 60743716.2.0000.5152). Ten non-carious human third molars were collected, cleaned, and examined via stereo microscopy for caries and structural defects. The teeth were stored in deionized water at 4 °C, which was changed weekly, for up to three months after extraction.^4,5,10,11^ The teeth were divided into two groups (n=5): control/non-irradiated and irradiated. To ensure the reliability and validity of the sample size, a power analysis (ANOVA) was conducted using SigmaPlot version 12.0 (Systat Software, San Jose, CA, USA). The power analysis for the effect sizes was based on FTIR results, which was considered the primary methodology of this study. As shown in Table 2, the mean difference was set at 0.006, representing the Carbonate/Phosphate (C:M ratio) difference between the control and irradiated groups with a standard deviation of 0.002. The power analysis revealed an effect size of 0.989, indicating the sample size provided sufficient power to detect significant differences.

**Table 2:** Means and standard deviation for the ratios of the bands corresponding to the chemical component in the FTIR spectra.

	M:M ratio Phosphate/Amide I	C:M ratio Carbonate/phosphate	Amide I/ Amide III	Amide I/CH2
Control	9.55 (1.78)A	0.028 (0.001)A	7.35 (0.46)A	9.60 (1.06)A
Irradiated	8.44 (0.95)A	0.022 (0.002)B	6.04 (0.66)B	9.51 (1.54)A

*Different letters show statistical difference in vertical

The irradiated group was exposed to a dose of 70 Gy from three-dimensional (3D) conformal radiotherapy using a linear accelerator (Clinac 600C; Varian Medical Systems, Palo Alto, CA, USA). This dose was delivered in daily fractions of 2 Gy, five days per week, over a period of seven weeks. During irradiation, the teeth were immersed in deionized water, which was changed weekly.^5^

### Specimen preparation

**Figure 1:**
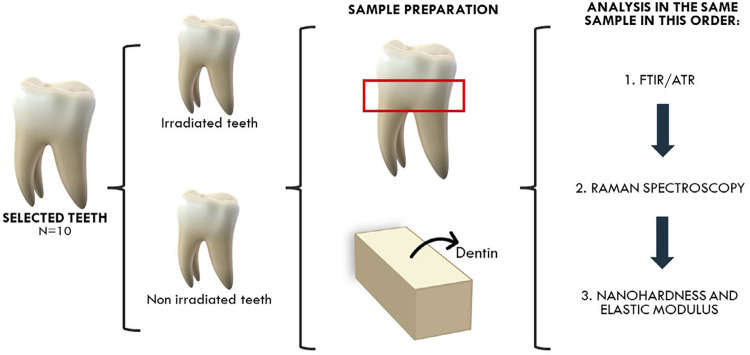
Illustrative scheme of specimen preparation.

The teeth were sectioned using a water-cooled diamond saw (IsoMet 1000; Buehler, Lake Bluff, IL, USA) under refrigeration. The first cut was made at the cementoenamel junction, followed by a cut 2.5 mm below it to obtain the cervical region of the root dentin. The enamel was then removed and all slices were cut longitudinally in the mesiodistal direction, resulting in two halves: buccal and lingual. Analyses were performed on the cementoenamel junction surface of the buccal halves with perpendicular dentin tubules. All tests were performed on the same sample. First, Fourier transform infrared spectroscopy (FTIR) and Raman spectroscopy were conducted, followed by instrumented nanoindentation. Figure 1 summarizes the sample preparation and methodologies.

### FTIR

The chemical composition of each sample was evaluated using FTIR (IR Vertex 70; Bruker, Ettlingen, Germany) with attenuated total reflectance (ATR). Each testing surface was positioned against the diamond crystal of the ATR unit, and constant pressure was applied to ensure contact. The absorbance spectrum was acquired by scanning the specimens 32 times in the range from 400 to 4,000 cm^1^ at a 4-cm^1^ resolution and analyzed using the OPUS 6.5 software (Bruker Optics, Billerica, MA, USA). After baseline correction and normalization, the FTIR spectra were analyzed using the following parameters: mineral/matrix ratio (M:M), expressed by the ratio of the integrated areas of band phosphate *v*_*1*_*, v*_*3*_ stretching mode – 960 and 1,040 cm^-1^, and protein amide I – 1,655 cm^-1^; carbonate/mineral ratio (C:M), the ratio of the integrated areas of carbonate *v*_*2*_ at 872 cm^-1^ to the phosphate *v*_*1*_, *v*_*3*_; amide I/amide III ratio, the ratio of the integrated areas of amide I at 1,655 cm^-1^ to amide III at 1,235 cm^-1^; and amide I/CH_2_ ratio, the ratio of the integrated areas of amide I at 1,655 cm^-1^ to the CH_2_ scissoring at 1,450 cm^-1^

### Raman spectroscopy

**Figure 2:**
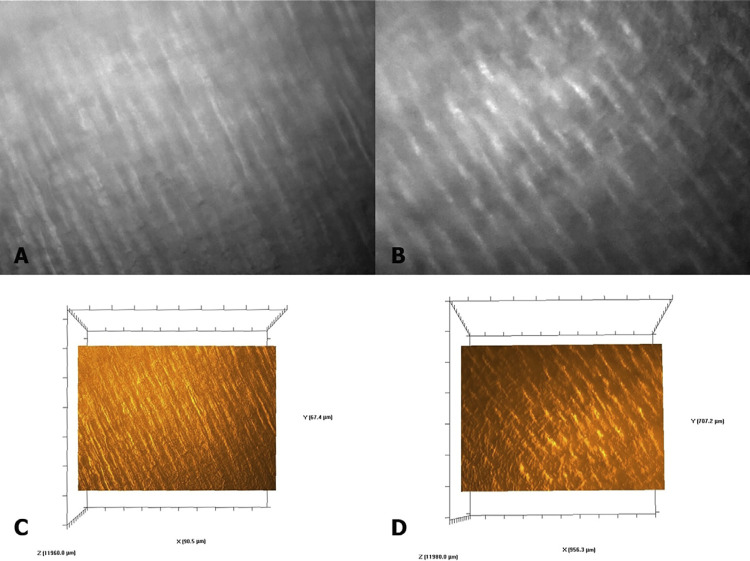
Images obtained using the optical microscope coupled with the Raman spectrometer, allowing the identification of the areas where the spectra were acquired, are shown in Figure 1A (control dentin) and 1B (irradiated dentin). The topographic images of the analyzed areas, generated by the LabSpec 6 software, are presented in Figure 1C (control dentin) and 1D (irradiated dentin).

Raman spectra were obtained using a LabRam HR Evolution Raman spectrometer (Horiba LabRam, Villeneuve d’Ascq, France) with an excitation power of 20 mW from a helium-neon (He-Ne) laser (632.8 nm). The Raman signal was acquired using a grid of 600 lines/mm centered between 300 and 3,100 cm^-1^ with a 400-μm confocal hole. The same analyzed regions in the FTIR spectra were evaluated using Raman spectroscopy. The surface was carefully demarcated using an optical microscope Olympus BX41 (Olympus Corporation, Tokyo, Japan) equipped with a 10X and 100X objective lens, adapted to the Raman spectrometer (Figure 2 A-B). The LabSpec 6 *software* (Horiba LabRam, Villeneuve d’Ascq, France) integrated with the Raman spectrometer, includes the advanced 3D Surface and volume display module, which enables volume data acquisition, full 3D rotation, filtering, transparency, and controls for displaying topographic images (Figure 2 C-D). The “flight mode” allows the image to be viewed from any orientation and angle.

OriginPro 7.5 software (OriginLab Corporation, Northampton, MA, USA) was used for spectral construction and analysis. The Raman spectra were adjusted by manual correcting the multiple baselines. The band at 960 cm^-1^ phosphate vibration v_*1*_ was selected as the internal standard for normalization. Peaks at 1,655/1,667, 1,246/1,270, and 1,450 cm^-1^ correspond to amide I, amide III, and CH_2_, respectively. This analysis identified the molecular conformation of the polypeptide chains.^13,14^ The mineral components in hydroxyapatite, the peak at 1,070 cm^-1^ attributed to the *v*_*1*_ vibration of the carbonate group, and the peak at 960 cm^-1^ attributed to the *v*_*1*_ vibration of the phosphate were also evaluated.^13,14^

Based on the Raman spectra, the proportion of the bands of phosphate v_1_ to amide I was calculated to analyze the differences in the mineral/matrix ratio (M:M) of the specimens. The proportions of carbonate at 1,070 cm^-1^ and phosphate at 960 cm^-1^ were obtained to analyze differences in mineral composition (C:M). To determine the nature of collagen in the specimens, the following indices were calculated: amide I/amide III for collagen organization and amide I/CH_2_ for change in collagen quality.^13^

### Nanohardness and elastic modulus

The specimens were embedded in polystyrene resin (AM 190 resin; Aerojet, São Paulo, SP, Brazil) for polishing. The surfaces were polished with silicon carbide paper grit sizes #600, 800, 1,200, and 2,000 (Norton, Campinas, SP, Brazil) under constant water irrigation and then polished with felt discs and metallographic diamond pastes six, three, one, and ¼ μm grit (Arotec, São Paulo, SP, Brazil). They were washed with deionized water and cleaned ultrasonically in distilled water for five minutes between each polishing step. The nanohardness and elastic modulus were measured using an instrumented nanoindenter UNAT (ASMEC, Dresden, Germany) with a diamond pyramidal tip (Berkovich type). The area function was calibrated using fused silica and sapphire as reference samples. The maximum load of the indentation tests increased quadratically up to 15 mN, with a dwell time of 30 seconds. The average maximum depth was approximately 1 mm. Indentations were performed at 12 locations with dentin tubules, 50 µm apart, to ensure measurements were unaffected. By monitoring the load, penetration depth, and surface elastic recovery after unloading, the nanohardness and elastic modulus were calculated using InspectorX software (ASMEC, Dresden, Germany) and the Oliver-Pharr method.^15^

### Scanning electron microscopy (SEM)

The samples were fixed in 2% glutaraldehyde solution in cacodylate buffer (Merck, Darmstadt, Germany) for two hours, dehydrated in increasing concentrations of ethanol (30%, 50%, 75%, 80%, 90%, 95% and 100%) (Êxodo Científica, Sumaré, SP, Brazil), immersed in hexamethyldisilazane (HMDS) (Merck, Darmstadt, Germany) for 10 min and kept in an incubator for 24 hours.^10^ Subsequently, they were fixed on stubs with a double-sided adhesive carbon tape (Electron Microscopy Sciences, Washington, PA, USA) and were sputter-coated with gold in a vacuum metallizing machine (Balzers SDC 050; Oerlikon Balzers, Balzers, Liechtenstein) and examined with a scanning electron microscope (Tescan VEGA 3 LMU; Tescan Orsay Holding, Brno, Czech Republic).

### Statistical analysis

Data were tested for normal distribution (Shapiro-Wilk, p>0.05) and equality of variances (Levene’s test, p>0.05). The chemical and mechanical properties were analyzed with a t-test considering the irradiation factor. SigmaPlot version 12.0 (Systat Software, San Jose, CA, USA) was used for analysis, with α<0.05 considered statistically significant.

## Results

### ATR/FTIR

Table 1 lists the means of the integrated areas of each band in the FTIR spectra. The irradiated group had higher values for amide I, amide III, CH_2,_ and phosphate, but lower values for carbonate. Table 2 shows the means and standard deviations of the FTIR ratio parameters. The *t*-test revealed no statistical differences for the M:M ratio (p=0.255) and the amide I/CH_2_ ratio (p=0.918) for the irradiated factor. However, for the C:M ratio a significant difference was found (p=0.004), with lower values in the irradiated group. Similarly, the amide I/amide III ratio showed a statistical difference (p=0.007), with lower values in the irradiated group.

**Table 1:** Mean of the integrated areas of each band corresponding to the chemical component in the FTIR spectra.

	Amide I	Amide III	CH2	Phosphate	Carbonate
Control	1.43	0.19	0.15	13.35	0.37
Irradiated	1.62	0.25	0.17	13.62	0.31

### Raman spectroscopy

Table 3 lists the means of the integrated areas of each band evaluated by Raman spectroscopy. For amide I and amide III, the irradiated group showed higher values compared to the control group. Phosphate and carbonate had the same values for control and irradiated groups. Table 4 shows the means and standard deviations of the ratios of the parameters. The *t*-test showed no significant difference for the C:M ratio (p=0.987). The M:M ratio revealed a significant difference (p<0.001), with lower values for the irradiated group. A significant difference was found between the control and irradiated groups for the amide I/amide III (p<0.001), and the irradiated group showed higher values compared to the control group. For the amide I/CH2 ratio, *t*-test had a statistical difference (p<0.001) for the irradiation factor, with higher values for the irradiated group.

**Table 3:** Means of the integrated areas of each Raman band corresponding to the chemical component in the Raman spectra.

	corresponding to the chemical component in the Raman spectra				
	Amide I	Amide III	CH2	Phosphate	Carbonate
Control	0.07	0.05	0.07	1	0.17
Irradiated	0.12	0.07	0.06	1	0.17

**Table 4:** Means and standard deviation for the ratios of the of the bands corresponding to the chemical component in the Raman spectra.

	M:M ratio Phosphate/Amide I	C:M ratio Carbonate/phosphate	Amide I/ Amide III	Amide I/CH2
Control	14.28 (1.38)A	0.175 (0.004)A	1.29 (0.10)B	0.98 (0.014)B
Irradiated	8.33 (0.63)B	0.175 (0.009)A	1.71 (0.16)A	2.10 (0.12)A

*Different letters show statistical difference in vertical

### Nanohardness and elastic modulus

Table 5 lists the mean values and standard deviations of nanohardness and elastic modulus. The irradiated group showed significantly lower mean nanohardness values than the control group (p=0.04). Similarly, the elastic modulus was higher in the control group. The t-test showed a significant difference for the elastic modulus (p=0.003) between the control and irradiated groups.

**Table 5:** Means and standard deviation for Nanohardness and Elastic Modulus.

	Nanohardness (GPa)	Elastic modulus (GPa)
Control	0.59 (0.02)A	20.6 (0.6)A
Irradiated	0.51 (0.1)B	17.8 (1.3)B

*Different letters show statistical difference in vertical

### Scanning electron microscopy (SEM)

**Figure 3:**
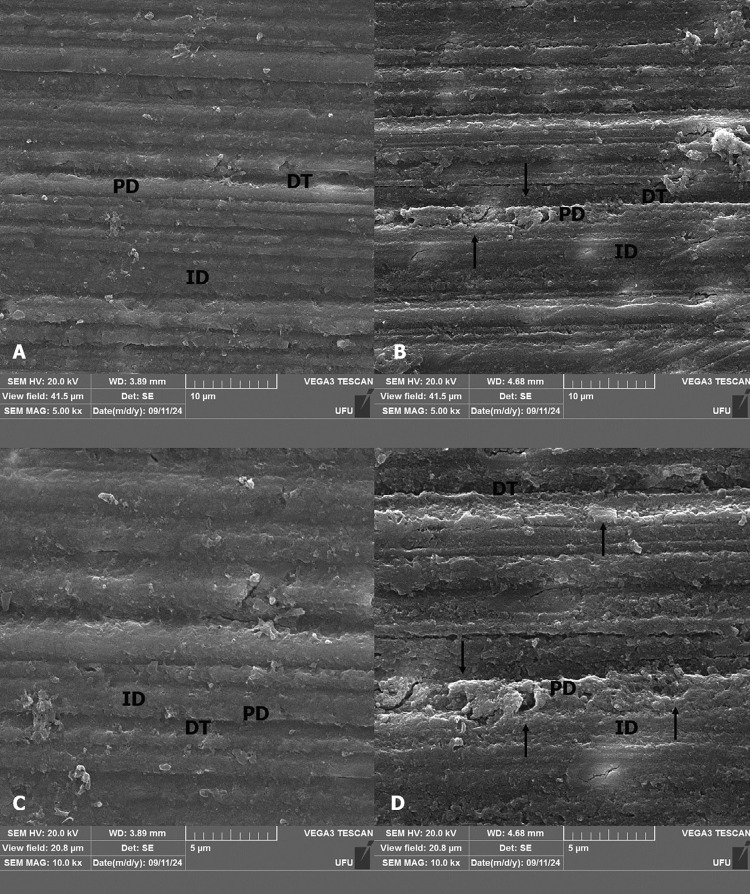
SEM images of the dentin. The images were obtained via scanning electronic microscopy at 5.00 kx (A, B) and 10.0 kx (C, D) magnifications. A, C – sound dentin (control). B, D – irradiated dentin. It is possible to note normal organization of dentin (A, C). In the irradiated dentin, the presence of inter (ID) and peritubular dentin (PD) is not clear, and there is no limit between the two structures (B, D). The arrows indicate the discontinuity between peri and intertubular dentin, with some points of destruction and break between them.

SEM images reveal that sound dentin (control) had a normal tissue organization (Figure 3 A and C), as evidenced by the presence of dentinal tubules (DT), peritubular dentin (PD) and intertubular dentin (ID). In the DT, the presence of PD is not clear, and there is not a limit between the two types of dentin (Figure 3 B and D). While some regions maintain a limit between the peri and intertubular dentin, there is a disruption and discontinuity between these structures, characterized by points of destruction and break between them.

## Discussion

The null hypotheses were rejected because radiotherapy changed the chemical composition and also the mechanical properties of the cervical dentin. It indicates mechanical weakening of the cervical region, which clinically manifests as vulnerability of the dental structure, potentially leading to total loss of the crown. Carious lesion in this region starts from the buccal surface, extend to the lingual surface, and progresses around the tooth as an annular lesion.^16^Previous studies have shown that oral cancer patients who were exposed to radiotherapy develop post-radiation dental lesions, initiating with enamel shear fractures that can result in partial to total enamel delamination.^7,17^ This enamel loss may be related to collagen alteration in irradiated teeth, indicating an instability mechanism of the DEJ.^18^ The results of this study confirmed the direct damage to the cervical region caused by radiotherapy, as evidenced in previous studies.^6,19,20^A significant reduction in microhardness values was observed in the irradiated dentin compared to the dentin that was not irradiated, which did not receive any treatment. This further highlights the essential role of fluoride in preserving the microhardness of irradiated dentin, as it has been shown to promote remineralization and mitigate the loss of hardness induced by irradiation.^21^

FTIR and Raman spectroscopic techniques were used for chemical analysis based on the vibrational modes of the compounds observed in the spectra. Both techniques can evaluate organic and inorganic compounds, but they differ in their methods for detecting chemical molecules and bonds.^18^ FTIR is based on light absorption, providing a small and well-controlled penetration depth by measuring the specimen layer in contact with the ATR crystal.^22^ This technique relies on the absorption of infrared radiation, promoting the transition from a low-energy vibrational state to a higher-energy state.^18^ Nevertheless, Raman spectroscopy is based on light scattering by matter, in which a change in the polarization of the molecules is observed when a visible or ultraviolet photon interacts with the vibrating molecular bonds, gaining or losing some energy, thus generating the spectrum.^18^ In Raman spectroscopy, the spectral analysis is performed in light scattering and reflection modes, enabling the analysis of samples at greater depths.^22^ Therefore, both techniques are complementary.^4^

In the FTIR spectra, significant changes were observed in the inorganic portion of the dentin. The irradiated group showed significantly lower values for the C:M ratio due to the smaller area of the carbonate band compared to the control group. The carbonate/phosphate band ratio indicates the extent of carbonate incorporation into the hydroxyapatite lattice and whether phosphate is replaced by carbonate.^23^ The results showed a decrease in this ratio, indicating that radiotherapy affected the mineral matrix. The literature suggests carbonate may replace phosphate ions in dentin hydroxyapatite, forming carbonated hydroxyapatite,^24,25^ which can explain the lower values observed for the carbonate band, as it causes deformations in the hydroxyapatite crystalline lattice, resulting in less stable and more acid-soluble phases.^4,26^ The radiation altered the mineralization pattern of dentin hydroxyapatite, affected the collagen structure, elevating amide I, which increases the concentration of organic components and reduced dentin’s resistance to demineralization.^4^Moreover, alterations in carbonate lead to deformations in the crystalline lattice of dentin, resulting in less stable and more acid-soluble phases, which may increase the tooth’s susceptibility to carious processes.^4^

Regarding changes in the organic matrix, FTIR and Raman spectroscopy showed higher values for the band areas of amide I and amide III in the irradiated groups. Both techniques showed statistical significance for the amide I/amide III band ratio. Similar results were observed for coronal and root dentin after radiotherapy.^4,27,28^ This ratio is associated with collagen organization.^13^ Amide I has the most intense absorption range in proteins, primarily governed by the stretching vibrations of the C=O (70-85%) and C–N (10-20%) groups.^14^ Amide III is a complex band, dependent on the details of the force field, side chains, and hydrogen bonding.^14^

Raman spectroscopy revealed greater changes, with a higher percentage difference between the integrated areas of the control and irradiated groups. Since the analysis was performed on the same sample, larger differences could be detected due to the greater depth achieved. This difference in molecule detection caused the ratios to behave differently, but both techniques indicated changes in collagen organization. Free radicals that were formed by radiotherapy break down the side hydrogen bonds of the collagen molecule when in the presence of water, altering its conformation.^29^ The free radicals can further interact with the terminal molecules in the collagen structure, causing structural rearrangement and increasing the amounts of amide I and amide III.^4^ Raman spectroscopy also revealed significant alterations in the amide I/CH_2_ and M:M band ratios, related to collagen quality, which occurred due to the greater area of the amide I and CH_2_ bands in the irradiated group. An increase in the band areas can indicate altered collagen quality induced by aging, hydration/dehydration, or radiological damage.^13,17^

The chemical analysis confirmed the fragility of dentin in the cervical region. Changes in the organic portion alter the conformation of collagen molecules and contribute to dentin degradation. Additionally, changes in the organic matrix may be related to the activation of collagen-degrading metalloproteinases.^30^ Although it was not the objective of this study, the literature reports that radiotherapy can activate collagenolytic enzymes, contributing to the progression of radiation-related caries.^30^

The results found in this study are consistent with others that have reported a decrease in dentin hardness during radiotherapy.^7,9,19,31^ High-energy irradiation can alter the mineral and organic matrices, with severity increasing at higher radiation dosage.^7,31^ Those studies associate the reduction in nanohardness after radiotherapy with collagen degradation, micromorphological changes, and the formation of less-structured hydroxyapatite crystals.^7^ Such changes indicate decreased microhardness values due to the direct effects of radiation on the oral structure^7,9,19,20,31^, with no preventive treatments applied to the dentin. This lack of intervention highlights the role of fluoride, as it has been shown to be effective in preserving microhardness values and reducing the effects of irradiation on dentin.^6,21^

In an oral environment highly prone to demineralization, characterized by reduced salivary flow and poor hygiene practices, the irradiated dentin appears to be more softened.^32,33^ Therefore, preventive strategies, such as oral hygiene practices and fluoride use, may promote an increase in microhardness values in irradiated dentin. Gel fluoride is recommended for post-radiotherapy patients. However, acidulated fluoride does not prevent the softening of microhardness in irradiated dentin due to the lack of saliva, which can exacerbate demineralization,^6^ whereas neutral fluoride has been shown to be effective in preserving microhardness values in irradiated dentin, as neutral fluoride treatments result in a greater increase in microhardness.^21^

Nanohardness and elastic modulus reflect the complex interactions of the tissue microstructure. Human dentin arrangement is responsible for mechanical properties such as tensile strength and viscoelasticity of the ﬁbrillar matrix.^34^ Since this study evaluated the chemical composition and mechanical properties of the same sample, it can be stated that the alteration of the hydroxyapatite mineralization pattern, along with structural changes in the collagen molecule, caused a reduction in nanohardness and elastic modulus values. Radiotherapy causes decarboxylation of the carboxylate side links in collagen, which are responsible for the interaction of the mineral matrix with hydroxyapatite crystals.^29^ The decrease in mechanical properties can also be related to the reduced mineral-organic interaction.

Scanning electron microscopy images have a different appearance between the irradiated and the control group. However, qualitative analyses indicate the potential effects of irradiation on dentin are not responsible for initial demineralization under clinical conditions, and oral care strategies can effectively control the development of radiation-related caries.^35^ In the control group, the dentin tissue appears homogeneous and displays a rougher, collagen-rich texture. In contrast, the SEM images of the irradiated dentin display a discontinuity of the limit between peritubular and intertubular dentin, with evidence of destruction and breaks between these regions.^27^The alterations observed in the dentin tissue following irradiation can be attributed to the reorganization of the collagen structure, as confirmed by FTIR and Raman analyses.^27^ This reorganization also impacts the mechanical properties of the dentin.

The results of this study are significant as they demonstrate the vulnerability of cervical dentin. Chemical alterations may be associated with the disorganization of apatite crystals,^31^ along with changes observed in collagen proteins molecules (amide I, II, and III).^4^ Such changes are directly related to dental biomechanical behavior, as shown by the significant reduction in nanohardness and elastic modulus. The mechanical changes reflect the direct damage from radiotherapy, weakening the dental element and contributing to early dental fracture and loss.

As limitations, the teeth were irradiated *in vitro* to evaluate the effects on the chemical composition and mechanical behavior of dentin without interference from factors such as salivary pH, xerostomia, and patients’ diet.^19^ Additionally, obtaining *in vivo* irradiated teeth that are sound and free of caries or restorations is challenging. Therefore, the use of *in vitro* irradiation enabled a more focused analysis of the direct effects of radiation on dental tissue. However, it limits the interference of oral factors, commonly found clinically in irradiated patients, and it does not consider the potential of fluoride and oral hygiene in preventing the destruction of irradiated dental structure.

Comprehending radiation-induced alterations in the cervical region is crucial to explain the clinical effects seen in post-radiation teeth. An understanding of changes caused by radiotherapy in the cervical region *in vitro* provides a basis for future studies. Some studies have examined external factors in the oral environment, such as patients’ diets and oral pH levels. Such studies, conducted *in situ* or using *in vivo* samples, have demonstrated changes that more accurately reflect in clinical conditions.^4,6,33,34,36^ Given their relevance, further longitudinal and clinical trials could be conducted to establish preventive and restorative protocols.

## Conclusions

Radiotherapy induced significant changes in dentin composition and mechanical properties, characterized by increased organic content and phosphate levels, reduced carbonate, and decreased nanohardness and elastic modulus. The findings highlight the adverse effects on dentin’s structural integrity.
